# Breast and pleuropulmonary metastasis of multirecurrent scalp dermatofibrosarcoma protuberans: a case report

**DOI:** 10.1186/s13256-017-1257-8

**Published:** 2017-04-08

**Authors:** N.-A. Ouédraogo Nde, N. A. Ouédraogo, H. A. Bambara, B. M. A. Tiemtoré-Kambou, F. Traoré, N. Zongo, M. S. Ouédraogo, G. P. Tapsoba, Achach Thouraya Chtioui, A. Lamien/Sanou

**Affiliations:** 1 Hôpital de District de Bogodogo, Service de Radiologie et d’Imagerie Médicale, Ouagadougou, Burkina Faso; 2 Unité de Recherche en Sciences de la Santé, Université Ouaga I Professeur Joseph Ki-Zerbo, Ouagadougou, Burkina Faso; 3 Service de Dermatologie, Centre Raoul Follereau, Ouagadougou, Burkina Faso; 4 Service d’Oncologie Médicale, Centre Hospitalier Universitaire Yalgado Ouedraogo, Ouagadougou, Burkina Faso; 5 Service de Dermatologie, Centre Hospitalier Régional Ouahigouya, Ouagadougou, Burkina Faso; 6 Service de chirurgie viscérale et oncologique, Centre Hospitalier Universitaire Yalgado Ouedraogo, Ouagadougou, Burkina Faso; 7 Service de Dermatologie, Centre Hospitalier Universitaire Yalgado Ouedraogo, Ouagadougou, Burkina Faso; 8 Laboratoire d’Anatomie Pathologie et de Cytologie à Monastir, Monastir, Tunisia; 9 Service d’Anatomopathologie, Centre Hospitalier Universitaire Yalgado Ouedraogo, Ouagadougou, Burkina Faso

**Keywords:** Metastasis, Breast, Pleuropulmonary, Secondary location, Burkina Faso

## Abstract

**Background:**

Dermatofibrosarcoma protuberans is a rare tumor, representing about 0.1% of skin malignant tumors. It is characterized by local aggressiveness with significant potential for recurrence. Although metastasis is rare, it does occur. We report a case of a Burkinabe woman with dermatofibrosarcoma protuberans.

**Case presentation:**

A 27-year-old Burkinabe woman consulted our institution for a recurrent scalp nodule that had been evolving for 13 years. At clinical examination, she was in good condition with a dry cough. An atrophic scarring alopecic plaque of 15-cm diameter in the right parietal region of the scalp, topped by an erythematous firm nodule measuring 3 × 2 × 2 cm, was noted, as well as a mobile nodule located in the axillary tail of the right breast. Cerebral computed tomodensitometry had not objectified the reach of the vault or the brain. A thoracic scan revealed four intrathoracic tissue masses straight to pleural touch. There were no evolutionary lesions in the abdominopelvic region. Histopathologic examination of the biopsy of the scalp nodule showed a proliferation of fibrous background, with fusiform cells carrying a storiform appearance. These cells had dark, elongated nuclei and showed some mitosis without atypia. The cells expressed CD34 intensely and diffusely. The test results were negative for PS100 and smooth muscle actin. The breast nodule showed the same profile as the scalp nodule.

**Conclusions:**

We concluded on the diagnosis of scalp dermatofibrosarcoma protuberans with breast metastasis and probable pleuropulmonary metastasis.

## Background

Dermatofibrosarcoma of Darier and Ferrand, or *dermatofibrosarcoma protuberans* (DP), is a low-grade fibrohistiocytic tumor characterized by a high rate of local recurrence [1]. It is a rare tumor, representing 0.1% of malignant skin tumors [2]. It usually affects adults between 20 and 50 years of age and is located on the trunk and proximal limbs [3, 4]. The diagnosis of this tumor is often delayed because of its various clinical presentations. It is characterized by local aggressiveness with significant potential for recurrence; although metastasis is rare, it does occur.

We report a case of a patient with DP of the scalp diagnosed after seven recurrences and six undocumented surgeries. Its uniqueness lies in the discovery of a breast nodule in the right axillary tail as well as intrathoracic masses in a 27-year-old woman.

## Case presentation

A 27-year-old Burkinabe woman from the north of Burkina Faso had consulted for a recurrent scalp nodule that had been evolving for 13 years. The scalp nodule had relapsed for the seventh time 6 months ago after six surgeries. It began as a painless nodule of the scalp that would have benefited from iterative surgical resections without histologic analysis.

The patient presented to our institution complaining of a dry cough lasting for 1 month. She was afebrile and in good condition. We noted an atrophic, indurated, scarring alopecic plaque measuring 15 cm in diameter in the center of her scalp, topped by a firm erythematous nodule measuring 3 × 2 × 2 cm in projection of the right parietal region. The nodular mass was slightly movable to the deep plane and painless.

**Fig. 1:**
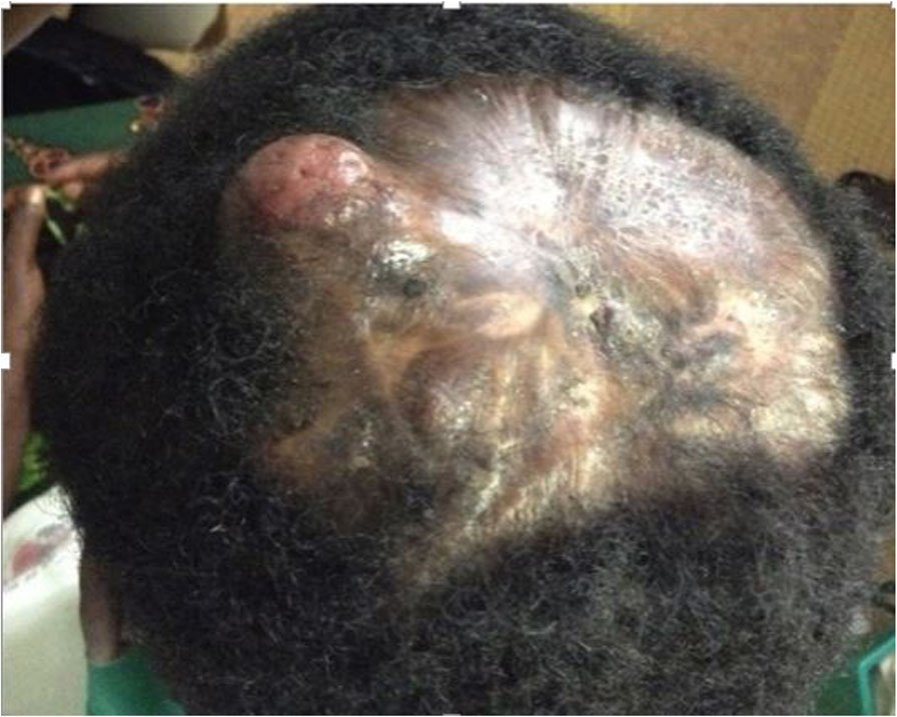
Tumor nodule in alopecic scarring atrophic plaque of the scalp

Three other nodules were also observed that were more palpable than visible, each measuring 1 cm in diameter, on the indurated alopecic plaque (Fig. 1). The patient’s physical examination revealed a painless mobile nodule located in the axillary tail of the right breast. The result of the patient’s respiratory system examination was normal. The results of the rest of her physical examination were normal.

**Fig. 2:**
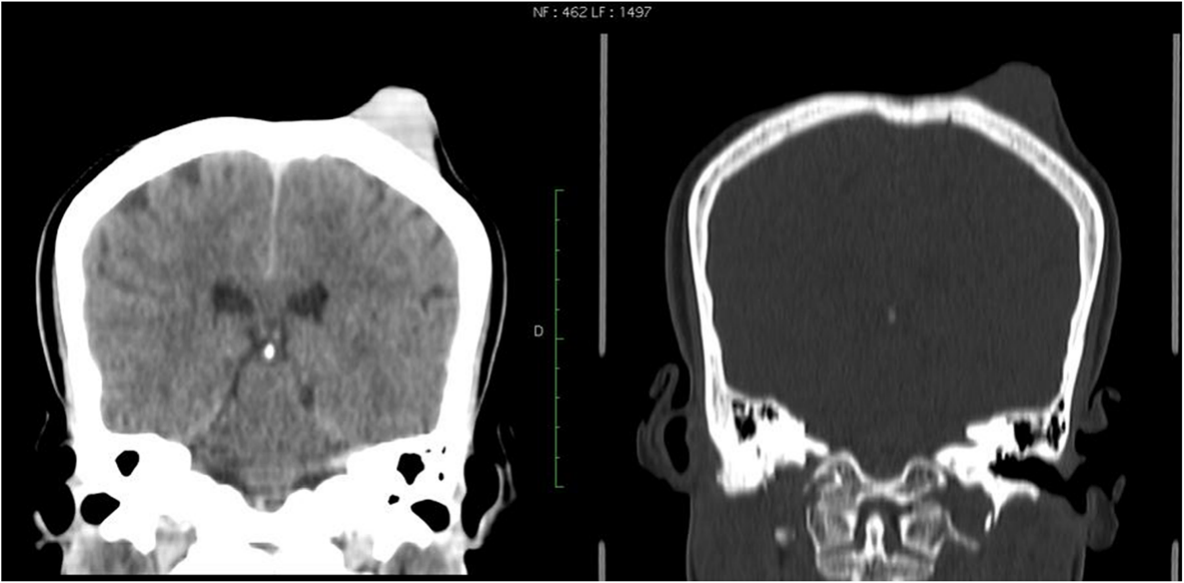
Coronal reconstruction of cerebral computed tomodensitometric scans showing the right parietal exophytic nodule without bone involvement

Cranioencephalic computed tomodensitometry (CT) was performed, which showed a soft tissue mass in the exophytic right parietal skin, measuring 30 × 24 × 17 mm, enhanced after iodine contrast agent injection. There were no signs of damage to the cranial vault or the brain (Fig. 2).

**Fig. 3:**
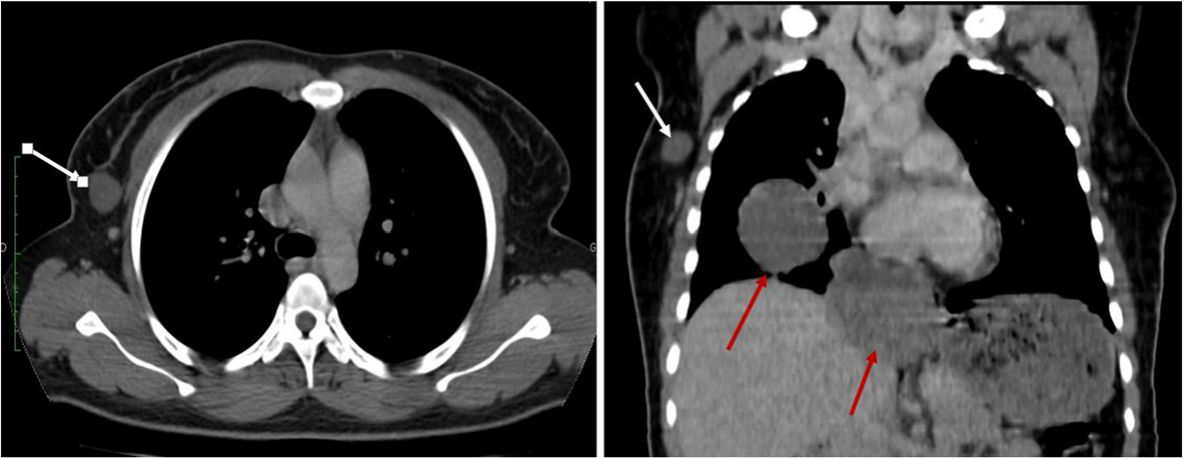
Coronal reconstruction of thoracic computed tomodensitometric scans showing the right breast nodule (*white arrow*) and thoracic extension pleural nodules (*red arrows*) in touch

Thoracic CT revealed four intrathoracic tissue masses that were straight to pleural contact and a nodule of the axillary tail of the right breast, measuring 23 × 22 × 18 mm (Fig. 3). The breast and chest nodules had the same characteristics before and after iodine contrast agent injection. There were no evolutionary lesions in the abdominopelvic region. Ultrasound of the breast nodule revealed an oval hypoechoic formation with regular contours. The result of an abdominopelvic ultrasound was normal.

A histologic study of the scalp nodule biopsy showed a proliferation of fibrous background fusiform cells carrying a storiform pattern. These cells had elongated, dark nuclei and showed some mitosis without atypia (three mitoses per 10 high-power fields at × 40 magnification). An immunohistochemical study demonstrated that the cells expressed CD34 intensely and diffusely, but the result was negative for PS100 (Fig. 5) and smooth muscle actin.

**Fig. 4:**
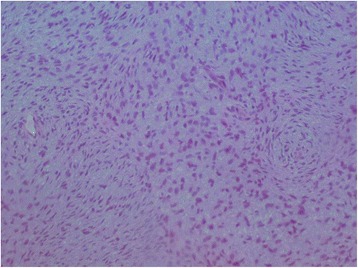
Histologic specimen of the breast nodule showing a storiform architecture. There is no atypia and no mitosis

**Fig. 5:**
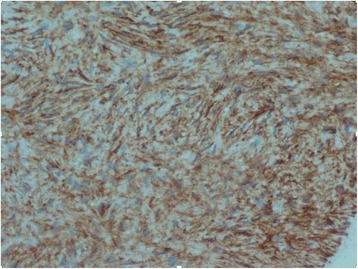
Histologic specimen of the breast nodule showing intense attachment of CD34

**Fig. 6:**
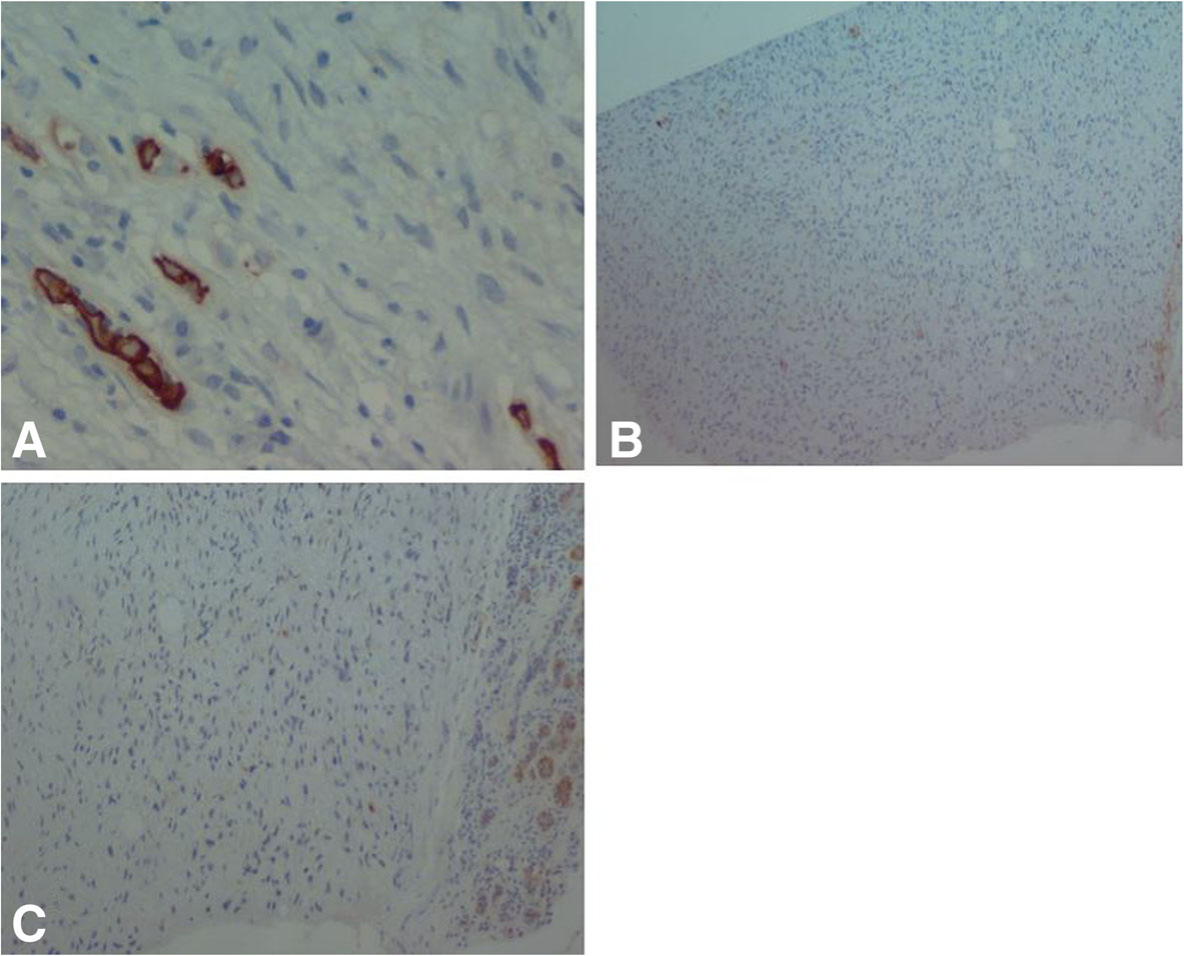
Immunohistochemical examination of the breast nodule. There is no attachment of cytokeratin (**a**), PS100 (**b**), or smooth muscle actin (**c**)

Histologic and immunohistochemical study of the breast nodule revealed the presence of a breast mesenchymal tumor (Fig. 4) with the same characteristics as the scalp nodule, and the result was positive for CD34 (Figs. 5 and 6). The combination of clinical, imaging, histologic, and immunohistochemical findings led us to a diagnosis of scalp DP with breast metastasis and probably pleuropulmonary metastasis. Surgery and chemotherapy with imatinib are being considered for our patient. ​﻿﻿Af﻿ter three rounds of chemotherapy, the breast and chest nodes partially regressed in size.﻿

## Discussion

DP was first described by Darier and Ferrand in 1924 as a progressive and recurrent dermatofibroma [5]. This tumor is characterized by slow evolution with a high propensity for local relapse. Our patient was experiencing a seventh recurrence. Its location on the scalp is uncommon. Indeed, the craniofacial location represents about 10% to 15% of the occurrences [6]. Despite its multiple relapses and disease duration of 13 years in our patient, we have not found any signs of local bone or intracranial extension. This was not the case in a report by Abe *et al*. [7], who found that DP recurred 7 years after three surgical resections of nodules of the scalp, brain invasion, and superior sagittal sinus.

DP metastasis is extremely rare. When it occurs, the preferred location is the lung [4]. The other secondary locations described are ganglion, brain, pancreas, liver, testicle, orbit, muscle, and the abdominopelvic region. Lymph node and visceral metastasis occurs in 1% and 4% of cases, respectively [8]. Chryssogonidis *et al*. [9] described pulmonary, brain, and testicular locations; Mahajan *et al*. [10] reported brain, muscular, and pleural locations; Yokoyama *et al*. [11] reported pancreatic and muscular locations; Gupta *et al*. [12] described liver and cerebral locations; Nakra *et al*. [13] reported an orbital location; and Berbis *et al*. [14] described lung, abdominal, and pelvic locations. A breast secondary location, as in the case of our patient, is extremely rarely reported in the literature. Pleuro-pulmonary nodules were probably metastatic even if they have not been proven histologically.

Because of the secondary locations of DP, sarcomatous transformation must be discussed. Indeed, regional and general metastases are also disclosed in the context of sarcomatous processing. This sarcomatous transformation can occur in 10% to 20% of DP cases. Histologic and immunohistochemical criteria of sarcomatous changes are cellular atypia, a fascicular pattern, ten mitoses per 10 high-power fields at × 40 magnification, decreased expression of CD34, and increased MIB-1 (EB1) with more than 5% of tumor tissue transformed. None of these histologic or immunohistochemical signs for sarcomatous transformation were found in our patient.

The differential diagnosis can also include neurofibroma. Immunohistochemical techniques help in the differential diagnosis. DP tumor cells express CD34 but not PS100 and factor VIIIa, in contrast to diffuse neurofibroma, which expresses PS100. This typical aspect of DP was noted in scalp and breast nodule samples from our patient. Our patient had DP of the scalp with breast metastasis and probable pleuropulmonary metastasis.

The management of DP is surgery. The recommended surgery is wide excision, removing the tumor *en bloc* with wide safety margins of 3 to 5 cm of healthy tissue. This is the only means of therapy that has demonstrated effectiveness in preventing recurrence. In another surgical method, the Mohs micrographic technique, the entire tumor is removed while a maximum of healthy tissue is conserved using extemporaneous three-dimensional intraoperative pathological control [15]. This technique could not be applied in our patient. However, chemotherapy with imatinib is used for locally advanced and metastatic DP, and it is associated with a 50% response rate [16]. Some authors recommend radiotherapy to reduce the risk of local recurrence, especially in cases of tumor recurrence, invasion of tumor margins, or in locations limiting large resections. Radiotherapy is not available in our country, so chemotherapy with imatinib and surgery with a wide excision were considered for our patient.

In general, the survival rate for DP is 99% at 5 years when there is no metastasis [17]. The prognosis depends on the quality of the surgical excision, local recurrence, and the occurrence of metastasis. For our patient, the prognosis is reserved because of the multirecurrence of breast and pleuropulmonary metastasis. The 5-year survival in these recurrent and metastatic cases is on the order of 12% [17].

## Conclusions

DP is a rare tumor characterized by a local malignant potential. Recurrence is common. The occurrence of secondary locations is very rare. A breast metastasis is also extremely uncommon; hence, the interest of our observations related to breast and pleuropulmonary metastases from scalp DP in a young woman of Burkina Faso.

## Abbreviations

CT: Computed tomodensitometry
DP: Dermatofibrosarcoma protuberans
